# Peer Review of “Machine Learning–Based Prediction of COVID-19 Mortality With Limited Attributes to Expedite Patient Prognosis and Triage: Retrospective Observational Study”

**DOI:** 10.2196/34083

**Published:** 2021-10-15

**Authors:** Victor Hugo Moquillaza Alcántara

**Affiliations:** 1 Data Management Area Asociación Via Libre Lima Peru

**Keywords:** COVID-19, coronavirus, medical informatics, machine learning, artificial intelligence, dimensionality reduction, automation, model development, prediction, hospital, resource management, mortality, prognosis, triage, comorbidities, public data, epidemiology, pre-existing conditions

*This is a peer-review report submitted for the paper “Machine Learning–Based Prediction of COVID-19 Mortality With Limited Attributes to Expedite Patient Prognosis and Triage: Retrospective Observational Study.”*

## Round 1 Review

### General Comments

This paper [1] shows useful information that would allow better health management in countries with a high incidence of COVID-19 cases. The rationale for the study is clear, but there is little scientific literature to support the information presented.

### Specific Comments

#### Major Comments

1. The introduction of the paper presents only 4 bibliographic references, which is scarce to defend the problem and justification of a study. In the summary, he mentioned at the beginning that human resources in hospitals are scarce, which is an important reality that has not been addressed in the introduction of the paper. I suggest starting by evaluating the problem of hospital saturation, with epidemiological indicators from various studies that can support this information (this will notably increase the number of references); then, justify the study with the potential benefits of using these types of tools.

2. During the discussion, technical aspects of the statistical models used are evaluated; however, I suggest that a comparison or appreciation can be provided regarding the utility and impact that these results would show in public health.

#### Minor Comments

3. In the Abstract, it is suggested that the general objective of the study be reported. Remember that the summary seeks to capture the reader’s attention and not saturate them with details.

4. In the first line of the introduction of the study, it says “... development of the COVID-19”; it should say “... development of the Coronavirus Disease (COVID-19)”. Remember that the first time an acronym is mentioned, its full name must be written.

5. According to the International Committee of Biomedical Journal Editors, the table description should be at the top of the table. In Tables 1, 2, and 3, the description is below.

6. In order that the tables and figures do not leave doubts to the readers, I suggest that in Table 2, there should be a footnote where it is specified what the author refers to with “AUC.”
